# The Chinese Association for the Study of Pain (CASP): Consensus on the Assessment and Management of Chronic Nonspecific Low Back Pain

**DOI:** 10.1155/2019/8957847

**Published:** 2019-08-15

**Authors:** Ke Ma, Zhi-Gang Zhuang, Lin Wang, Xian-Guo Liu, Li-Juan Lu, Xiao-Qiu Yang, Yan Lu, Zhi-Jian Fu, Tao Song, Dong Huang, Hui Liu, You-Qing Huang, Bao-Gan Peng, Yan-Qing Liu

**Affiliations:** ^1^Department of Algology, Xinhua Hospital, Shanghai Jiaotong University School of Medicine, Shanghai, China; ^2^Department of Algology, The Second Affiliated Hospital of Zhengzhou University, Zhengzhou, Henan, China; ^3^Department of Algology, Affiliated Hospital of Guizhou Medical University, Guiyang, Guizhou, China; ^4^Pain Research Center, Department of Physiology, Zhongshan School of Medicine, Sun Yat-Sen University, Guangzhou, China; ^5^Department of Algology Medicine, Nanjing Drum Tower Hospital, The Affiliated Hospital of Nanjing University Medical School, Nanjing, China; ^6^Department of Algology, The First Affiliated Hospital of Chongqing Medical University, Chongqing, China; ^7^Department of Algology, Xijing Hospital, Fourth Military Medical University, Xian, China; ^8^Department of Algology, Shandong Provincial Hospital Affiliated to Shandong University, Jinan, Shandong, China; ^9^Department of Algology, The First Affiliated Hospital of China Medical University, Shenyang, China; ^10^Department of Algology, The Third Xiangya Hospital, Central South University, Changsha, China; ^11^Department of Algology, West China Hospital of Sichuan University, Chengdu, China; ^12^Department of Algology, The Second Affiliated Hospital of Kunming Medical University, Kunming, Yunnan, China; ^13^Department of Spinal Surgery, General Hospital of Armed Police Force, Beijing, China; ^14^Department of Algology, Beijing Tian Tan Hospital, Capital Medical University, Beijing, China

## Abstract

Chronic nonspecific low back pain (CNLBP) is defined as pain or discomfort originating from the waist, which lasts for at least 12 weeks, but no radiculopathy or specific spinal diseases. CNLBP is a complicated medical problem and places a huge burden on healthcare systems. Clinical manifestation of CNLBP includes discogenic LBP, zygapophyseal joint pain, sacroiliac joint pain, and lumbar muscle strain. Further evaluation should be completed to confirm the diagnosis including auxiliary examination, functional assessment, and clinical assessment. The principle of the management is to relieve pain, restore function, and avoid recurrence. Treatment includes conservative treatment, minimally invasive treatment, and rehabilitation. Pharmacologic therapy is the first-line treatment of nonspecific LBP, and it is most widely used in clinical practice. Interventional therapy should be considered only after failure of medication and physical therapy. Multidisciplinary rehabilitation can improve physical function and alleviate short-term and long-term pain. The emphasis should be put on the prevention of NLBP and reducing relevant risk factors.

## 1. Overview

Low back pain (LBP), defined as pain or discomfort in the area between the lower ribs and the gluteal folds, is a common and potentially debilitating condition with or without leg pain. Chronic low back pain (CLBP) refers to low back pain lasting for more than 12 weeks. Patients with LBP can be placed into one of three categories, i.e., nonspecific low back pain, low back pain associated with radiculopathy or spinal stenosis, and low back pain associated with specific spinal disease [1]. Chronic nonspecific LBP (CNLBP) is defined as pain and discomfort lasting for at least 12 weeks, but no radiculopathy, specific spinal disease, or nerve root pain. The current practice of diagnosis and treatment of LBP is often empirical, but not based on scientific methodology. It is time for our experts to convene, discuss, and formulate consensus for the diagnosis and treatment of CNLBP in China. It is also necessary to refer to existing evidence on the topic and international consensus for the diagnosis and treatment of LBP. The knowledge in this field has been combined with the conclusions drawn from some good clinical studies carried out in China. We believe that the consensus will be of great significance for pain management in China and will be of great reference value for other national pain experts in the world.

## 2. Etiology and Epidemiology of CNLBP

### 2.1. Common Causes of CLBP

Based on etiology, CLBP is often classified into two categories: specific and nonspecific CLBP. Specific CLBP has obvious causes such as infection, tumor, fracture, or inflammatory disease. However, 80%–90% of CLBP is nonspecific, intractable, and difficult to cure [1–4]. It is a great challenge to clarify the specific causes of CLBP. With the development of technologies and diagnostic tests (local anesthetic injection or discography), etiologic factors can be identified in 90% of patients with CLBP [5]. A study by DePalma et al. [5] on patients with nonspecific CLBP showed that prevalence of zygapophyseal joint pain, sacroiliac joint pain, and discogenic pain was 31%, 18%, and 42%, respectively.

### 2.2. Discogenic LBP

Discogenic LBP is not caused by lumbar intervertebral disc herniation. It is defined as pain caused by changes in the internal structure of lumbar discs, despite intervertebral discs of normal morphology. The pathologic feature is the formation of zones of vascularized granulation tissue with extensive innervation in fissures extending from the outer part of the annulus to the nucleus pulposus [6]. Peng [7] divided discogenic LBP into two types based on discography findings: internal annular disruption (IAD) and internal endplate disruption (IED).

### 2.3. Zygapophyseal Joint Pain

Zygapophyseal joint pain is identified as pain arising from any structures of the lumbar facet joints, including bony articulations, hyaline cartilage surfaces, the synovial membranes, and the fibrous capsules [8, 9].

### 2.4. Sacroiliac Joint Pain

Sacroiliac joint pain is defined as pain originating from the region of the sacroiliac joint, exacerbated by stress and provocation tests, and relieved by sacroiliac joint injections with local anesthetic [10].

### 2.5. Soft Tissue-Derived LBP

Soft tissues such as ligaments and muscles around the lumbar spine play an important role in maintaining the position of the body, as well as enhancing the stability, balance, and flexibility of the spine. Diseases of the soft tissues such as ligaments, fascia, and muscles can produce pain, which is called muscle pain clinically.

### 2.6. Epidemiology of CNLBP

CNLBP is a serious medical and social problem, which often leads to the loss of labor force. Although many of the studies in the literature have focused on the incidence and prevalence of CNLBP, there is a lack of accurate data on the incidence of the disease because the consensus on its definition has not been established so far [11]. In 2012, a systematic survey of the global adult population showed that the point prevalence, 1-month prevalence, 1-year prevalence, and lifelong prevalence of CNLBP were 12%, 23%, 38%, and 40%, respectively [12–18]. In China, CNLBP is the most common disease in Departments of Orthopedics, Rehabilitation Medicine and Pain Medicine, accounting for 1/3 of daily outpatient visits, second only to upper respiratory tract infections. At present, the medical cost for CNLBP is greater than that of coronary heart disease, diabetes, arthritis, and cerebrovascular disease [19, 20]. CNLBP is no longer a simple medical problem, and it can cause complicated psychological problem and serious social medical economic burden on patients [21–24].

## 3. Pathogenesis of CNLBP

A large number of basic and clinical studies have shown that CNLBP presents not only nociceptive pain, but also neuropathic pain, usually accompanied by central and peripheral sensitization [25, 26].

### 3.1. Discogenic LBP

The painful disc is characterized histologically by formation of a zone of vascularized granulation tissue in the posterior part of the disc, with infiltration of macrophages and mast cells [27]. Macrophages are not only the main phagocytic cells in the inflammation but also secrete a large number of growth factors and cytokines. The aggregation of mast cells in the painful disc may be closely related to the formation of new blood vessels in the disc and the fibrosis of the disc tissue [7]. Meanwhile, discogenic LBP has also the characteristic of visceral pain [6, 28]. At present, the main pathological features of discogenic LBP are the annulus fibrosus tearing, vascularized granulation tissue gradually growing from the outer part of the annulus into the nucleus pulposus and the degenerative annulus fibrosus, and nucleus pulposus releasing inflammatory mediators. These inflammatory mediators sensitize nociceptors, which causes LBP [27]. Internal endplate disruption is a form of discogenic LBP. Endplate injury can be subdivided into two types: formation of Schmorl's nodules and endplate degeneration. The endplate is richly innervated and the nerve density of endplate is similar to that of annulus fibrosus, which strongly suggests that the lesion in endplate is an important source of LBP [7, 29–31].

### 3.2. Zygapophyseal Joint Pain

Chronic inflammation caused by degeneration, repetitive stress, and/or cumulative low-level trauma leads to facet joint hyperplasia, joint effusion, and joint capsule dilatation, which stimulates nerve terminals distributed on the facet joints and thereafter produces pain response [8]. The development of zygapophyseal joint pain is often insidious with common predisposing factors including lumbar spondylolisthesis, intervertebral disc degeneration, and old age [9].

### 3.3. Sacroiliac Joint Pain

Sacroiliac joint pain can be divided into intra-articular causes (infection, spondyloarthropathy, and arthritis) and extra-articular causes (fracture, myofascial pain, enthesopathy, and ligament injury) [10]. The mechanism of sacroiliac joint pain is considered as the combination of axial loading and rotation [32]. The risk factors are leg length discrepancy, abnormal gait, scoliosis, trauma, sacral fixation after lumbar spinal fusion surgery, heavy manual labor, and pregnancy [10]. Histopathology reveals that there are abundant nociceptors and proprioceptors in the sacroiliac joint capsule, ligament, and subchondral bone of sacroiliac joint, which indicated that any injury to surrounding tissues could cause pain [33–36].

### 3.4. Soft Tissue-Derived LBP

Muscle imbalance, muscle spasm, and muscle contracture represent a three-part pathological response resulting in chronic soft tissue-derived back pain [37]. Chronic accumulation of soft tissue injury renders muscles too weak to maintain the normal function position of the waist, which strains the deep ligament [38]. Insufficiency of circulation originating from peripheral nerves and blood vessels in the muscle which are pressed, together with metabolite accumulating and inflammatory substances, forms a new point of pain and even leads to muscle atrophy and fibrosis [39]. Often ligaments and muscles are shortened on one side and loose on the other side, which leads to posture imbalance and pain spreading [40].

## 4. Clinical Manifestation of CNLBP

### 4.1. Discogenic LBP

The most important clinical feature of discogenic LBP is poor tolerance to sitting position, which often exacerbates the pain. Most patients suffer from repeated LBP attacks, aggravated by fatigue, long standing time, exposure to cold, and coughing or sneezing. Pain is relieved in bed. The pain is mainly located in the waist, and sometimes it radiates to the lower extremities. The pain can be below the knee. There is no specific sign or symptom for its diagnosis. The symptoms also include lower limbs numbness, coldness, and intermittent claudication.

### 4.2. Zygapophyseal Joint Pain

Zygapophyseal joint pain cannot be relieved by rest. The symptoms include low back pain, soreness, and stiffness in the morning. It can be relieved after moderate activity, but it is aggravated by excessive activity. The pain worsens at night. The pain can be relieved when lying flat or by massage. There is no obvious tenderness in the lumbar region.

### 4.3. Sacroiliac Joint Pain

Sacroiliac joint pain is moderate to severe pain on one side of the low back radiating to the hip or groin area. The patient is often unable/reluctant to walk or grudgingly limp. The pain can be alleviated by bending hips on bed. Serious patients cannot turn over in bed.

### 4.4. Lumbar Muscle Strain

Lumbar muscle strain is diffuse and dull pain in the muscles on both sides of the waist and above the iliac crests. It tends to get worse in the morning and can be alleviated with some exercise, but too much exercise can aggravate the condition. When the patient is resting, the condition is often relieved and becomes worse when the patient is tired. Pain becomes more acute with bending posture and can be relieved by stretching the back or tapping on the waist. The pain caused by lumbar muscle strain can spread to the buttocks or thighs, but not to the lower leg or foot. This pain can be associated with symptoms of autonomic nervous system disorders such as cold limbs, visceral pain, etc. Sitting tolerance decreases in the patients.

## 5. Signs

1. Changes in physiological curvature of spine: scoliosis, decreased or lost physiological curvature, and kyphosis.
2. Lumbar spinal range of motion: normal lumbar range of motion includes 75–90 degrees of flexion, 30 degrees of extension, 20–35 degrees of left and right side bending, and 90 degrees of rotation unilaterally. The range of motion is reduced in CLBP, and the activity in all directions of the lumbar spine is limited.
3. Physical examination reveals local or extensive tenderness or percussion pain on the low back.
4. Magnetic resonance imaging (MRI) or computed tomography (CT) scan does not display nerve root compression.
5. Neurological examination usually does not show abnormalities in sensory, motor, and tendon reflexes.

## 6. Diagnosis and Differential Diagnosis

Diagnosis of CNLBP represents a challenge for clinicians because of the complexity of its etiology, which involves physical, mental, and psychosocial factors. The following are the steps to take for the diagnosis and differential diagnosis of CNLBP (Table 1).

## 7. History and Physical Examination

The first step for clinical evaluation of patient includes a focused medical history and physical examination. Clinicians should inquire about the location and quality of pain as well as the stability or progression of pain and neurological symptoms (e.g., sensory and motor dysfunction). Clinicians should also inquire about history of malignancy or tuberculosis, history of previous treatment, and response to the treatment. Special attention should be paid to the patients with risk factor associated with LBP, as well as the patients with previous symptom. Clinicians should help patients with LBP enter 1 of 3 broad categories based on a focused medical history and physical examination: nonspecific LBP, LBP potentially associated with radiculopathy or sciatica, or LBP potentially associated with another specific spinal cause (red flag). It is important to conduct differential diagnosis for nonspecific LBP. The history should include assessment of psychosocial risk factors. The evaluation of LBP is shown in Table 2.

## 8. Auxiliary Examination

Further evaluation (e.g., imaging and electrophysiology) may be required to confirm the diagnosis. Imaging test includes plain radiography, CT, and MRI. Clinicians should not take imaging tests as routine in patients with LBP for initial evaluation. Diagnostic imaging tests should only be performed for LBP patients when severe or progressive neurologic deficits are presented or serious underlying conditions being suspected on the basis of history and physical examination, such as lumbar disc herniation or spinal stenosis which are potential candidates for surgery [41–43].

## 9. Functional Assessment

Functional assessment of nonspecific LBP can be achieved by valid questionnaires. Chinese version of Roland-Morris Disability Questionnaire (MDQ) and Oswestry Disability Index (ODI) is being researched to test its reliability and validity. The Chinese version of the Fear-Avoidance Belief Questionnaire (FABQ-CHI) has proved valid to evaluate the aspect of pain, health, and dysfunction in CNLBP patients. Moreover, the intelligent device for energy expenditure and activity (IDEEA) and surface electromyography (sEMG) can also be used for the functional assessment of patients with nonspecific LBP objectively.

## 10. Clinical Assessment

Once the diagnosis of CNLBP is made, the attending physician should conduct a clinical assessment. The goal of the evaluation is to define the cause of the disease, set up appropriate treatment strategies, and assess the efficacy of treatment objectively later. The principle of clinical assessment should be standard, quantitative, comprehensive, and dynamic [44]. The assessment includes the location, quality, intensity, frequency, and duration. Physician should also evaluate the mental and psychosocial factors of patients [45]. The method of evaluation consists of self-assessment and Behavior Rating Scale (BRS). For the assessment of pain level, Visual Analogue Scale (VAS), Numerical Rating Scale (NRS), and pain questionnaires can be adopted. Functional assessment is usually achieved using ODI, Quebec Back Pain Disability Scale (QBPDS) [46], Short Form (36) Health Survey, and Roland-Morris Disability Questionnaire (RMDQ) [47]. One way to evaluate the back muscle function objectively and in real time is to apply surface electromyography (sEMG) [48]. Physicians can evaluate the psychosocial factors through Fear-Avoidance Belief Questionnaire (FABQ) and Tampa Scale for Kinesiophobia (TSK).

## 11. Treatment of CNLBP

### 11.1. Principle of Treatment

**Figure 1:**
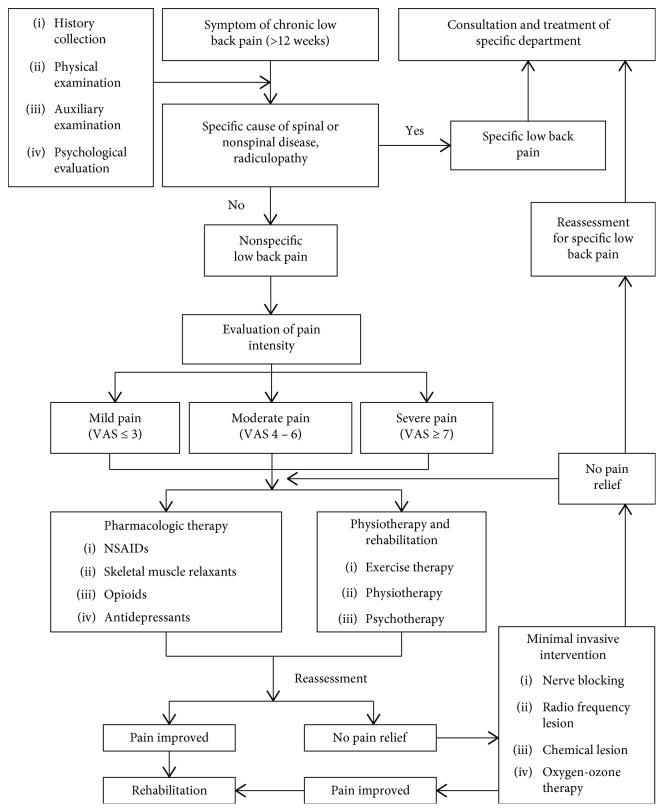
Flow chart of the treatment.

The aim of the treatment is to relieve pain, restore function, and avoid recurrence. Treatment includes conservative treatment, minimally invasive treatment, and rehabilitation. Surgical treatment is not introduced here.  Figure 1 is the flow chart of the treatment.

## 12. Pharmacologic Therapy

Pharmacologic therapy is the first-line treatment of nonspecific LBP, and it is most widely used in clinical practice. Physicians should formulate individualized treatment plans according to each patient's condition and modify them if necessary. The combination of different treatments may improve the clinical efficacy.

### 12.1. Nonsteroidal Anti-Inflammatory Drugs (NSAIDs)

NSAIDs can provide reliable pain relief for the patients with CNLBP [49–51]. Taking more than one NSAID at the same time is not recommended. If one NSAID prescription fails to provide sufficient pain relief within two weeks of treatment, the attending physician may consider a different kind of NSAIDs prescription for such patients. Physicians should also remain alert for the ceiling effect of NSAIDs. In addition, there is association between exposure to NSAIDs and increased risk for myocardial infarction [50, 52]. Clinicians should therefore assess cardiovascular and gastrointestinal risk factors before prescribing NSAIDs and recommend continuous taking of NSAIDs for no more than 3 months. Nonselective NSAIDs include ibuprofen, diclofenac, etc. Selective Cox-2 inhibitors include etoricoxib and celecoxib, which have the effect of alleviating gastrointestinal complications.

### 12.2. Skeletal Muscle Relaxants

Antispasmodic muscle relaxants play a role in reducing muscle spasm associated with lower back pain [53, 54]. The possible mechanism of antispasticity of *α* 2-adrenergic receptor agonist (e.g., tizanidine) is to increase the proportion of gamma-aminobutyric acid (GABA) compared to glutamate in presynaptic level [55]. Additionally, they may provide pain relief, antidepression, and gastrointestinal protection. Combination therapy of skeletal muscle relaxants and NSAIDs can reduce nonspecific LBP effectively and improve the motor function in general. The gastrointestinal protective effect of skeletal muscle relaxants can offset the gastrointestinal damage induced by other drugs [56]. The possible mechanism of antispasmodic action for eperisone is to inhibit the activity of gamma motor neuron [57]. Chlorzoxazone and baclofen are also commonly used for relieving the spasm of LBP patients.

### 12.3. Opioids

Use of opioids is an option for nonspecific LBP patients who are uncontrolled when other drugs are being treated [58–60]. Physicians should start with prescription of weak opioids with extended-release such as tramadol hydrochloride extended-release tablets [61, 62]. Patients should be maintaining the opioid intake for management of pain, not only when pain is severe [63]. Early use of opioids can prevent early pain, which makes early functional training possible. Also, one of the main purposes of opioids treatment in CLBP patients is to facilitate functional rehabilitation [64].

### 12.4. Antidepressants

Patients with chronic pain often suffer other affective disorders like depression and anxiety [65]. Use of antidepressants is an option for pain relief in patients with CLBP. Physicians can choose amitriptyline, duloxetine, or venlafaxine to attenuate the depression associated with chronic pain. To avoid the withdrawal symptoms of antidepressants, patients should withdraw from antidepressants gradually according to the prescription of physician [66–68].

### 12.5. Other Medications

85 percent of patients with LBP display hyperalgesia [69, 70]. CNLBP is often associated with neuropathic pain [4]. Use of anticonvulsants is effective treatment for the LBP patients with pain hypersensitivity. Gabapentin and pregabalin can restore the function of excitatory neurons by blocking the voltage-dependent calcium channel which can reduce the excitatory input [66, 71]. The dysfunction of sodium ion channel plays an important role in the development of chronic pain [69]. Bulleyaconitine provides sufficient relief of pain hypersensitivity induced by chronic pain by selectively blocking the overactive sodium ion channel [71]. Recent clinical data have demonstrated the effect of traditional patch, e.g., Tibetan medicine pain relief patch, relieving pain in muscles and joints [72, 73].

## 13. Minimally Invasive Interventional Therapy

The right diagnosis and precise identification of the pain generator is the prerequisite for a successful interventional therapy. Interventional therapy should be considered only after failure of medication and physical therapy. In order to give patients a precise and targeted treatment, ultrasound, X-ray, or CT-guided imaging is highly recommended for the intervention.

### 13.1. Epidural Injection

#### 13.1.1. Route of Epidural Injection

Epidural injections have been used extensively to treat back and leg pain. Among the routes of transforaminal, caudal, and interlaminar approaches, transforaminal epidural injections have gained wide acceptance for the treatment of lumbar and lower extremity pain. The potential advantage of transforaminal approach over interlaminar or caudal approach is targeted delivery of small volume steroid to the site of pathology, presumably to an inflamed nerve root [74].

#### 13.1.2. Drug Choices and Frequency for Epidural Injection

Drugs used in epidural injections are usually compounds of steroid and local anesthetics. Methylprednisolone acetate and betamethasone are recommended for the epidural injection [75]. As long-acting particulate steroid accidentally into bloodstream may cause serious complications, nonparticulate steroid is recommended [76]. As for the local anesthetics, 0.5% lidocaine or 0.25% bupivacaine is recommended. The recommended volume of liquid is 7–10 ml for interlaminar route, 1-2 ml for transforaminal route, and 20–50 ml for caudal route. For those patients getting incomplete pain relief from an epidural injection, repeated injections may be required with the interval of 1–3 weeks. No more than three procedures is recommended within one year [77].

### 13.2. Medial Branch Block

Medial branch nerve block is mainly applied as the diagnostic test for the facet joint-mediated LBP [9]. Each facet joint is innervated by the medial branches of the spinal nerve roots from the same level and upper level; therefore, the diagnostic test should be given at the two levels at the same time. To avoid false-positive results from the spread of the anesthetic into the epidural space, 0.5 ml of test volume is recommended [9].

### 13.3. Sacroiliac Joint Injection

Limited high-quality RCT data support the effectiveness of sacroiliac joint injection in the treatment of sacroiliac joint pain. It is mainly performed as the diagnostic test. Due to the irregular sacroiliac joint surface, the sacroiliac joint is difficult to be injected by palpation [78]. The success rate of palpation-guided sacroiliac joint injection was reported to be merely 12%. Therefore, imaging-guided injection is recommended [79]. The needle should be inserted into the inferior one third of the joint. As the joint cavity is small, no more than 1 ml of injectant is recommended [79].

### 13.4. Intradiscal Injection of Methylene Blue

Injection of methylene blue into the painful disc is a minimally invasive procedure for the treatment of discogenic LBP. However, there have been few reports [80, 81]. Diagnostic discography with positive result is required before proceeding with the treatment [81]. The recommended injectant is 1-2 ml of 1% methylene blue.

### 13.5. Radiofrequency Treatment for CNLBP

#### 13.5.1. Radiofrequency Ablation of Medial Branch of Spinal Nerve

Radiofrequency ablation of medial branch nerve is applied for the treatment of zygapophyseal LBP verified by positive diagnostic test. Moderate evidence exists for the short-term pain relief, but the effect after one year is controversial [82]. The puncture target is the junction of superior articular process and transverse processes. To avoid nerve root injury, the tip of the needle should be away from the intervertebral foramen under lateral fluoroscopy. Nerve stimulation test is essential during the procedure. During the stimulation process, pain within the target area should be elicited with sensory stimulation of less than 0.5 V. No lower limb muscle twitch is observed with motor stimulation of more than 1.0 V.

#### 13.5.2. Pulsed Radiofrequency of Dorsal Root Ganglion

Pulsed radiofrequency of dorsal root ganglia was used for treatment of radiating lower extremity pain. Several case reports have reported that the benefits of such treatment are short term, but rigorous evidence is lacking [83]. For those patients getting more than 50% of pain relief with pulsed radiofrequency of dorsal root ganglion, repeated procedures for 2–5 times may be considered. For those with less than 50% of pain relief, repeated procedures are not warranted. Local anesthesia is not recommended at the beginning of the procedure.

#### 13.5.3. Sacroiliac Joint Radiofrequency Ablation

Sacroiliac joint radiofrequency ablation is performed for treatment of sacroiliac joint pain. Diagnostic sacroiliac joint injection is required to confirm the pain generator. As sacroiliac joint is innervated by multiple lumbar and sacral nerves and the relevant nerves are diffusely distributed, bipolar radiofrequency ablation is recommended [84], although the related reports are scarce [85, 86].

#### 13.5.4. Low-Temperature Plasma Disc Decompression for Spinal Disc Herniation

The use of low-temperature plasma disc decompression to treat spinal radiculopathy due to a contained disc herniation is supported by prospective controlled trials [87]. The indication for plasma disc decompression is contained disc herniation causing LBP with or without radiating leg pain for more than 3 months, and the disc height reduction is less than 50% [88]. The contraindication of plasma decompression includes spinal disc prolapse, huge contained herniated disc with herniation taking over one third of sagittal distance of the spinal canal, herniated disc calcification, severe lumbar canal stenosis, lumbar instability, and neurologic function impairment [89].

#### 13.5.5. Percutaneous Intradiscal Radiofrequency Thermocoagulation

Percutaneous intradiscal radiofrequency thermocoagulation is a treatment option for contained herniated disc. But no evidence is available for the treatment of contained herniated lumbar disc. Different reports utilized different inclusion criteria and different parameter settings in radiofrequency procedures, and the reported efficacy differed dramatically [90].

### 13.6. Intradiscal Electrothermal Therapy (IDET)

IDET is mainly applied for low back pain originating from lumbar disc degeneration. However, the reported efficacy was mixed [91, 92]. It is recommended as a therapeutic option for recalcitrant patients with conservative treatment. Some of the literature demonstrated the efficacy of IDET for LBP superior to radiofrequency thermocoagulation [91].

### 13.7. Oxygen-Ozone Therapy

Oxygen-ozone therapy is widely applied in the context of pain management. Ozone may be injected into the spinal disc or the muscles, or administered through the transforaminal route [93]. Ozone can stimulate the repairing system of the body, activate inhibitory interneurons, and generate analgesic effects through the release of enkephalin and endorphin [94]. Ozone can also oxidize the proteoglycan of nucleus pulposus, destroy nucleus pulposus cells, and play an anti-inflammatory role [95]. It also generates analgesic effect by directly acting on the inflammatory tissues around the nerves [96]. The local oxidation and analgesic effect could result in muscle relaxation and vasodilatation and expedite metabolism of muscles [97]. The injection of ozone into the disc or the paravertebral space should be imaging-guided. 5–10 ml of ozone of 40∼60 *μ*g/ml is recommended for intradiscal injection, and 3∼5 ml of ozone of 35 *μ*g∼40 *μ*g/ml is recommended for trigger point injection. Trigger point injection could be performed at 3–5 points in one treatment. Four consecutive treatments could be repeated twice per week. The therapy is contraindicated for patients with hyperthyroidism and glucose-6-phosphate dehydrogenase deficiency [98].

### 13.8. Neuromodulation Techniques

#### 13.8.1. Spinal Cord Stimulation (SCS)

SCS is reported to be an effective treatment for failed back surgery syndrome (FBSS) and complex regional pain syndrome [99]. The effective rate was 50%–70% throughout follow-up period of 6 months to 2 years [100]. The treatment of lower extremity pain is better than that of LBP [101]. SCS is recommended to be the last resort for LBP patients who failed in various conservative therapies, epidural injection, endoscopic transforaminal discectomy, or even open surgery. Strict selection of patients is vital. All cases require SCS testing prior to permanent implantation. The test takes about 3–10 days.

#### 13.8.2. Intrathecal Drug Delivery Systems (IDDSs)

IDDS is applied mainly for cancer pain. Occasionally, it can be utilized for FBSS according to anecdotal case presentations reported [102]. Long-term usage of intrathecal drug might lead to uncertain efficacy, drug resistance, and complicated postoperative management; therefore, it is only recommended for FBSS and nonspecific LBP refractory to drug therapy, interventional treatments, or surgeries. Drugs frequently used in IDDS are opioids and local anesthetics. Other drugs such as baclofen and dexmedetomidine could also be used [103]. Trial administration of drug intrathecally is required to observe the analgesic effect and side effects before IDDS surgery.

### 13.9. Silver Needle Thermoconduction Therapy

Silver needle thermoconduction therapy is indicated for soft tissue originated pain, especially for myofascial pain syndrome in the lumbar and buttock region and muscle spasm [104]. In addition, silver needle thermoconduction therapy can also be applied for lumbar disc herniation, lumbar discogenic pain, and FBSS [105–107].

### 13.10. Acupotomy

Acupotomy is indicated for soft tissue originated pain. It was reported that acupotomy can effectively treat the third lumbar transverse process syndrome, iliolumbar ligament injury, and lumbar gluteal myofasciitis [108, 109]. In contrast to silver needle thermoconduction therapy, acupotomy is especially applicable to localized soft tissue originated pain [108, 110].

## 14. Rehabilitation

### 14.1. Exercise

Appropriate exercises can relieve pain to some extent. There is no evidence that exercise therapy improves dyskinesia in short term or medium term (6 months); however, there is evidence of significant improvement in the long term (over 12 months). Exercise therapy mainly includes pilates, tai chi, and yoga [111–113].

### 14.2. Acupuncture

Acupuncture can relieve pain immediately, and its effect can be maintained for up to 12 weeks. However, its long-term efficacy is unknown [114].

### 14.3. Massage

Massage can relieve the subacute and chronic LBP in short term and improve the patients' function. Massage combined with exercise can achieve better therapeutic effect.

### 14.4. Manipulation

Spinal manipulation therapy alleviates pain in a short time and improved the functional status of patients (1 month), but the long-term effect was poor [115].

### 14.5. Physical Therapy

#### 14.5.1. Ultrasound

Due to the inconsistency in evaluation methods, ultrasound has not been shown effectiveness on pain relief or functional improvement in the current literature [116].

#### 14.5.2. Transcutaneous Electrical Nerve Stimulation (TENS)

Systemic review found no significant difference between TENS and acupuncture in short-term or long-term improvement of pain [117, 118].

#### 14.5.3. Low-Level Laser Therapy

Compared with sham laser group, low-level laser therapy may relieve pain, but the effect is limited [118].

### 14.6. Lumbar Support Therapy

There is no evidence that lumbar support therapy is effective for LBP [119].

### 14.7. Traction Therapy

Compared with placebo, sham traction, or no traction, traction therapy has no significant effect on pain relief or improving physical function. However, this technique is effective for patients with radiation pain [120].

### 14.8. Psychological Therapy

The treatment of CNLBP cannot ignore psychological treatment. Progressive relaxation can relieve the posttreatment pain [121, 122] and promote functional improvement. Electromyographic biofeedback and operant therapy can relieve pain but is ineffective in improving physical function. Cognitive therapy for LBP has no significant benefits [123, 124].

## 15. Multidisciplinary Rehabilitation

Multidisciplinary rehabilitation includes physical, biological, psychological, and social therapy, involving experts from different disciplines. Studies have shown that multidisciplinary rehabilitation can improve physical function and alleviate short-term and long-term pain. With rehabilitation, patients may have the chance to resume their normal work [125].

## 16. Efficacy of Treatment Evaluation Criteria and Follow-Up

The efficacy should be evaluated at 2, 4, 8, and 12 weeks and 6 months after treatment. Evaluation indicators and tools include VAS, ODI, surface EMG, psychological assessment, spinal flexion and extension, shuttle walking test, and patient satisfaction and expectation [126]. The assessment should also include working state after back to work, sick leave days, medication, and side effects.

## 17. Prevention and Health Education

### 17.1. Risk Factors

There are many factors related to CNLBP, including age, occupation, psychological factors, heredity, sex, pregnancy, bodyweight, and unhealthy lifestyle. Some occupations (such as manual workers, typists, and taxi drivers), obesity, a sedentary lifestyle, and frequent bending are important risk factors for CNLBP. Psychological and genetic factors and pregnancy are closely related to the incidence of LBP [127] (Table 3).

### 17.2. Prevention

To prevent LBP, special attention should be paid to supporting the back, maintaining good posture, changing the habits which may lead to LBP, and maintaining ideal bodyweight [131].

  Protection measures: they include minimizing bending; adjusting the height of working chair to a suitable level to avoid bending; and when lifting, using leg strength as much as possible to reduce the stress on the waist [132].
  Sitting posture: it includes keeping waist straight, abdomen straight, and feet on the ground; using a soft cushion on your back to relax your lumbar muscles; and reducing tension.
  Sleeping posture: it includes lying on the side with knees bent and sleeping on a firm mattress with less cushioning to support a neutral-spine position (sleeping on a soft mattress may gradually deform lumbar spine curvature resulting in injury to lumbar muscles and soft tissue).
  Walking posture: it includes standing up straight, with chin up, eyes forward, without leaning forward or backward. Women with LBP should not wear high heels. High heels (>3 cm) can put a lot of stress on the waist and back muscles, increasing the risk of LBP.
  Obese patients should try to lose weight. Weight gain will bring burdens on the back. Pregnancy is also a heavy burden on the lumbar spine. Pregnant women should maintain correct posture and rest before giving birth.

## 18. Statement

1. Nonspecific low back pain has a long diagnostic cycle, high treatment difficulty, and high incidence.
2. Pathogenesis includes discogenic LBP, internal endplate disruption, zygapophyseal joint pain, sacroiliac joint pain, and soft tissue-derived LBP.
3. Auxiliary examination may confirm the diagnosis and is helpful for differential diagnosis.
4. Pharmacologic therapy is the first-line treatment. Individualized treatment plans should be formulated to obtain clinical efficacy.
5. Interventional therapy should be considered only after failure of medication and physical therapy which gives patients a precise and targeted treatment.
6. Rehabilitation includes exercise, acupuncture, massage, manipulation, lumbar support therapy, physical therapy, and psychological therapy and could relieve pain.
7. Disease prevention and health care can effectively improve the prognosis of CNLBP.

## Figures and Tables

**Table 1:** Differential diagnosis for nonspecific LBP.

Possible cause	Symptoms and physical examination	Imaging test	Laboratory test
Cancer	History of cancer, unexplained weight loss, and age >50 years	Lumbosacral plain radiography and MRI	ESR and tumor marker
Vertebral infection	Fever, history of recent infection, and tuberculosis	MRI	ESR, CRP, PPD, or PCT
Syndrome of cauda equina	Urinary retention, fecal incontinence, sensory disorder in saddle area, and motor deficits	MRI	None
Vertebral compression fracture	Older age, osteoporosis, and use of corticosteroids	Lumbosacral plain radiography, BMD and MRI	None
Ankylosing spondylitis	Morning stiffness, improvement after exercises, nocturnal pain, and younger age	Pelvis plain radiography	ESR, CRP, and HLA-B27
Radiculopathy	Progressive symptoms and motor weakness	CT or MRI	EMG and NCV
Symptomatic lumbar disc herniation	Back pain with leg pain in the distribution area of nerve root L4, L5, or S1 Positive for straight-leg-raise test Radicular pain present >1 month	None	None
Spinal stenosis	Older age, walking and standing worsen the symptom, pain relieved by sitting	CT or MRI	None

**Table 2:** Diagnostic protocol for nonspecific LBP.

Measures	Key points
(A) History inquiry	
Duration of LBP	Acute pain: within 4 weeks; subacute pain: 4 to 12 weeks; chronic pain: >12 weeks
Location of pain	Lumbosacral region
Characteristic of pain	Localized pain, radiating pain, burning sensation
Duration of pain attack	Consistent pain, intermittent pain, and night time episode
Sensory change	Numbness, stiffness, hypoesthesia, and noseresthesia
Other aspects	Education, occupation, BMI, infection, cancer, osteoporosis, endocrinopathy, history of trauma, and spine surgery
(B) Physical examination	
Inspection	Spine deformity, local condition
Palpation	Tenderness
Percussion	Percussion pain
(C) Accessory examination	
Signs	Lasegue test, Bragard sign, Gaenslen test, and Waddell test
Imaging test	Plain radiography, CT, and MRI
Electrophysiology	Electromyography and somatosensory evoked potential
Laboratory test	Erythrocyte sedimentation rate, C reactive protein, and HLA-B27

**Table 3:** Risk factors for CNLBP.

Risk factors	Description
Age [20]	Age is positively associated with the incidence.
Psychology [128]	Stress, anxiety, and depression may increase the incidence of LBP.
Occupation [129]	Long-term spinal heavy burden, excessive rotation, or vibration increases the risk of LBP; high-risk occupations are miners, drivers, farmers, and caregivers.
BMI [130]	Obesity is positively correlated with LBP incidence.
Gender	Women are more than men.
Genetics	LBP has familial aggregation.
Pregnancy	More than 50% pregnant women in the early pregnancy have LBP. This may be related to elevated levels of estrogen and progesterone.
Lifestyle	Smoking and sedentary lifestyle increase the risk of LBP.

## References

[1] Balagué F., Mannion A. F., Pellisé F., Cedraschi C. (2012). Non-specific low back pain. *The Lancet*.

[2] Lai J., Porreca F., Hunter J. C., Gold M. S. (2004). Voltage-gatedsodiumchannels andhyperalgesia. *Annual Review of Pharmacology and Toxicology*.

[3] Kallewaard J. W., Edelbroek C., Terheggen M., Raza A., Geurts J. W. (2019). A prospective study of dorsal root ganglion stimulation for non-operated discogenic low back pain. *Neuromodulation: Technology at the Neural Interface*.

[4] Baron R., Binder A., Attal N., Casale R., Dickenson A. H., Treede R.-D. (2016). Neuropathic low back pain in clinical practice. *European Journal of Pain*.

[5] DePalma M. J., Ketchum J. M., Saullo T. (2011). What is the source of chronic low back pain and does age play a role?. *Pain Medicine*.

[6] Bao-gan P. (2015). Progress in diagnosis and treatment of discogenic low back pain. *Chinese Journal of Pain Medicine*.

[7] Peng B.-G. (2013). Pathophysiology, diagnosis, and treatment of discogenic low back pain. *World Journal of Orthopedics*.

[8] van Kleef M., Vanelderen P., Cohen S. P., Lataster A., Van Zundert J., Mekhail N. (2010). 12. Pain originating from the lumbar facet joints. *Pain Practice*.

[9] Cohen S. P., Raja S. N. (2007). Pathogenesis, diagnosis, and treatment of lumbar zygapophysial (facet) joint pain. *Anesthesiology*.

[10] Vanelderen P., Szadek K., Cohen S. P. (2010). 13. Sacroiliac joint pain. *Pain Practice*.

[11] Andersson G. B. J. (1999). Epidemiological features of chronic low-back pain. *The Lancet*.

[12] Juniper M., Le T. K., Mladsi D. (2009). The epidemiology, economic burden, and pharmacological treatment of chronic low back pain in France, Germany, Italy, Spain and the UK: a literature-based review. *Expert Opinion on Pharmacotherapy*.

[13] Mousavi S. J., Akbari M. E., Mehdian H. (2011). Low back pain in Iran. *Spine*.

[14] Nascimento P. R. C. D., Costa L. O. P. (2015). Prevalência da dor lombar no Brasil: uma revisão sistemática. *Cadernos de Saúde Pública*.

[15] Gouveia N., Rodrigues A., Eusébio M. (2016). Prevalence and social burden of active chronic low back pain in the adult Portuguese population: results from a national survey. *Rheumatology International*.

[16] Wang S., Kou C., Liu Y. (2015). Rural-urban differences in the prevalence of chronic disease in northeast China. *Asia Pacific Journal of Public Health*.

[17] Yu D., Bain C., Williams G. (2012). A systematic review of the global prevalence of low back pain. *Arthritis & Rheumatism*.

[18] Garcia J. B., Hernandez-Castro J. J., Nunez R. G. (2014). Prevalence of low back pain in Latin America: a systematic literature review. *Pain Physician*.

[19] Maniadakis N., Gray A. (2000). The economic burden of back pain in the UK. *Pain*.

[20] Deyo R. A., Dworkin S. F., Amtmann D. (2014). Report of the NIH task force on research standards for chronic low back pain. *The Spine Journal*.

[21] Goertz Y., Abdi S. (2006). Diagnosis and minimally invasive treatment of lumbar discogenic pain ??? A review of the literature. *The Clinical Journal of Pain*.

[22] Manchikanti L., Singh V., Pampati V. (2001). Evaluation of the relative contributions of various structures in chronic low back pain. *Pain Physician*.

[23] Schwarzer A. C., Aprill C. N., Derby R., Fortin J., Kine G., Bogduk N. (1995). The prevalence and clinical features of internal disc disruption in patients with chronic low back pain. *Spine*.

[24] Rashbaum R. F., Ohnmeiss D. D., Lindley E. M., Kitchel S. H., Patel V. V. (2016). Sacroiliac joint pain and its treatment. *Clinical Spine Surgery*.

[25] Liu X. G., Zhou L. J. (2015). Long-term potentiation at spinal C-fiber synapses: a target for pathological pain. *Current Pharmaceutical Design*.

[26] Swayne L. A., Bourinet E. (2008). Voltage-gated calcium channels in chronic pain: emerging role of alternative splicing. *Pflügers Archiv—European Journal of Physiology*.

[27] Peng B., Wu W., Hou S., Li P., Zhang C., Yang Y. (2005). The pathogenesis of discogenic low back pain. *The Journal of Bone and Joint Surgery. British Volume*.

[28] García-Cosamalón J., Del Valle M. E., Calavia M. G. (2010). Intervertebral disc, sensory nerves and neurotrophins: who is who in discogenic pain?. *Journal of Anatomy*.

[29] Peng B., Chen J., Kuang Z., Li D., Pang X., Zhang X. (2009). Diagnosis and surgical treatment of back pain originating from endplate. *European Spine Journal*.

[30] Modic M. T., Masaryk T. J., Ross J. S., Carter J. R. (1988). Imaging of degenerative disk disease. *Radiology*.

[31] Lotz J. C., Ulrich J. A. (2006). Innervation, inflammation, and hypermobility may characterize pathologic disc degeneration. *The Journal of Bone and Joint Surgery-American Volume*.

[32] Cohen S. P., Chen Y., Neufeld N. J. (2013). Sacroiliac joint pain: a comprehensive review of epidemiology, diagnosis and treatment. *Expert Review of Neurotherapeutics*.

[33] Dreyfuss P., Henning T., Malladi N., Goldstein B., Bogduk N. (2009). The ability of multi-site, multi-depth sacral lateral branch blocks to anesthetize the sacroiliac joint complex. *Pain Medicine*.

[34] Szadek K. M., Hoogland P. V., Zuurmond W. W., de Lange J. J., Perez R. S. (2008). Nociceptive nerve fibers in the sacroiliac joint in humans. *Regional Anesthesia and Pain Medicine*.

[35] Foley B. S., Buschbacher R. M. (2006). Sacroiliac joint pain. *American Journal of Physical Medicine & Rehabilitation*.

[36] Simopoulos T. T., Manchikanti L., Singh V (2012). A systematic evaluation of prevalence and diagnostic accuracy of sacroiliac joint interventions. *Pain Physician*.

[37] Wang Xiang Z. M., Ren T., Feng S., Yang B., Ling D., Qu D. (2018). Comparison of clinical outcomes of modified posterior midline approach and traditional approach in the treatment of single-segment lumbar degenerative diseases. *Chinese Journal of Bone and Joint Surgery*.

[38] Bussières A. E., Stewart G., Al-Zoubi F. (2018). Spinal manipulative therapy and other conservative treatments for low back pain: a guideline from the Canadian chiropractic guideline initiative. *Journal of Manipulative and Physiological Therapeutics*.

[39] Stupar G. (1996). Low back pain: a twentieth century health care enigma. *Spine*.

[40] Harms-Ringdahl K., Ekholm J. (1986). Intensity and character of pain and muscular activity levels elicited by maintained extreme flexion position of the lower-cervical-upper-thoracic spine. *Scandinavian Journal of Rehabilitation Medicine*.

[41] Patrick N., Emanski E., Knaub M. A. (2016). Acute and chronic low back pain. *Medical Clinics of North America*.

[42] Golob A. L., Wipf J. E. (2014). Low back pain. *Medical Clinics of North America*.

[43] Webb R., Brammah T., Lunt M., Urwin M., Allison T., Symmons D. (2003). Prevalence and predictors of intense, chronic, and disabling neck and back pain in the UK general population. *Spine*.

[44] Huang Z., Ma J., Chen J., Shen B., Pei F., Kraus V. B. (2015). The effectiveness of low-level laser therapy for nonspecific chronic low back pain: a systematic review and meta-analysis. *Arthritis Research & Therapy*.

[45] Imamura M., Imamura S. T., Targino R. A. (2016). Paraspinous lidocaine injection for chronic nonspecific low back pain: a randomized controlled clinical trial. *The Journal of Pain*.

[46] Fregni C. M., Koppenaal T., Knottnerus J. A., Spigt M., Staal J. B., Terwee C. B. (2016). Measurement properties of the Quebec back pain disability scale in patients with nonspecific low back pain: systematic review. *Physical Therapy*.

[47] Chiarotto A., Maxwell L. J., Terwee C. B., Wells G. A., Tugwell P., Ostelo R. W. (2016). Roland-Morris disability questionnaire and Oswestry disability index: which has better measurement properties for measuring physical functioning in nonspecific low back pain? Systematic review and meta-analysis. *Physical Therapy*.

[48] Akbari M., Sarrafzadeh J., Maroufi N., Haghani H. (2015). Changes in postural and trunk muscles responses in patients with chronic nonspecific low back pain during sudden upper limb loading. *Medical Journal of the Islamic Republic of Iran*.

[49] Wong J. J., Côté P., Ameis A. (2016). Are non-steroidal anti-inflammatory drugs effective for the management of neck pain and associated disorders, whiplash-associated disorders, or non-specific low back pain? A systematic review of systematic reviews by the Ontario protocol for traffic injury management (OPTIMa) collaboration. *European Spine Journal*.

[50] Southerst L. (2003). Gastrointestinal effects of NSAIDs and coxibs. *Journal of Pain and Symptom Management*.

[51] van Tulder M. W., Scholten R. J. P. M., Koes B. W., Deyo R. A. (2000). Nonsteroidal anti-inflammatory drugs for low back pain. *Spine*.

[52] Goldstein J., Cryer B. (2015). Gastrointestinal injury associated with NSAID use: a case study and review of risk factors and preventative strategies. *Drug, Healthcare and Patient Safety*.

[53] Jones S. L., Henry S. M., Raasch C. C., Hitt J. R., Bunn J. Y. (2012). Individuals with non-specific low back pain use a trunk stiffening strategy to maintain upright posture. *Journal of Electromyography and Kinesiology*.

[54] Schinkel-Ivy A., Nairn B. C., Drake J. D. M. (2013). Investigation of trunk muscle co-contraction and its association with low back pain development during prolonged sitting. *Journal of Electromyography and Kinesiology*.

[55] Malanga G., Reiter R. D., Garay E. (2008). Update on tizanidine for muscle spasticity and emerging indications. *Expert Opinion on Pharmacotherapy*.

[56] Maeda-Hagiwara M., Watanabe H., Kanaoka R., Watanabe K. (1986). Influence of clonidine and a new related imidazoline derivative (tizanidine) on rat gastric mucosa. *Pharmacology*.

[57] Mehta S. G., Pawar D. R., Chandanwale A. S. (2011). Evaluation of eperisone hydrochloride in the treatment of acute musculoskeletal spasm associated with low back pain: a randomized, double-blind, placebo-controlled trial. *Journal of Postgraduate Medicine*.

[58] Cifuentes M., Webster B., Genevay S., Pransky G. (2010). The course of opioid prescribing for a new episode of disabling low back pain: opioid features and dose escalation. *Pain*.

[59] Furlan A. D., Sandoval J. A., Mailis-Gagnon A., Tunks E. (2006). Opioids for chronic noncancer pain: a meta-analysis of effectiveness and side effects. *Canadian Medical Association Journal*.

[60] Kalso E., Edwards J. E., Moore A. R., McQuay H. J. (2004). Opioids in chronic non-cancer pain: systematic review of efficacy and safety. *Pain*.

[61] Müller F. O., Odendaal C. L., Müller F. R., Raubenheimer J., Middle M. V., Kummer M. (1998). Comparison of the efficacy and tolerability of a paracetamol/codeine fixed-dose combination with tramadol in patients with refractory chronic back pain. *Arzneimittel-Forschung*.

[62] Schnitzer T. J., Gray W. L., Paster R. Z., Kamin M. (2000). Efficacy of tramadol in treatment of chronic low back pain. *The Journal of Rheumatology*.

[63] Hale M. E., Dvergsten C., Gimbel J. (2005). Efficacy and safety of oxymorphone extended release in chronic low back pain: results of a randomized, double-blind, placebo- and active-controlled phase III study. *The Journal of Pain*.

[64] Tao S., Zhi-Jian F., Wen-Ge S. (2012). Application of opioids in chronic non-cancerous pain. *Chinese Journal of Pain Medicine*.

[65] Xiao-Yan X., Juan Z., Li Z. (2016). Progress of the common mechanisms of pain and depression. *Chinese Journal of Pain Medicine*.

[66] Schnitzer T. J., Ferraro A., Hunsche E., Kong S. X. (2004). A comprehensive review of clinical trials on the efficacy and safety of drugs for the treatment of low back pain. *Journal of Pain and Symptom Management*.

[67] Salerno S. M., Browning R., Jackson J. L. (2002). The effect of antidepressant treatment on chronic back pain. *Archives of Internal Medicine*.

[68] Staiger T. O., Gaster B., Sullivan M. D., Deyo R. A. (2003). Systematic review of antidepressants in the treatment of chronic low back pain. *Spine*.

[69] Yan J., Zou K., Liu X. (2016). Hyperexcitability and sensitization of sodium channels of dorsal root ganglion neurons in a rat model of lumber disc herniation. *European Spine Journal*.

[70] Peng X., Wu N., Chen S.-Y., Yu X., Andrews J. S., Novick D. (2013). Utilization of duloxetine and celecoxib in osteoarthritis patients. *Current Medical Research and Opinion*.

[71] Yan-Qing L., Xiao-Ning D., Ying-de W. (2011). Clinical study of bulleyaconitine A tablets in common chronic pain Chinese. *Journal of Pain Medicine*.

[72] Maihöfner C., Schmelz M., Forster C., Neundörfer B., Handwerker H. O. (2004). Neural activation during experimental allodynia: a functional magnetic resonance imaging study. *European Journal of Neuroscience*.

[73] Xiao L., Qiang Y., Li W. (2013). Efficacy and safety of Chee-Zheng pain relieving plaster vs. muskiness plaster for musculoskeletal pain and swelling: a meta-analysis. *China Pharmacy*.

[74] Abdi S., Datta S., Trescot A. M. (2007). Epidural steroids in the management of chronic spinal pain: a systematic review. *Pain Physician*.

[75] Carette S., Leclaire R., Marcoux S. (1997). Epidural corticosteroid injections for sciatica due to herniated nucleus pulposus. *New England Journal of Medicine*.

[76] Pountos I., Panteli M., Walters G., Bush D., Giannoudis P. V. (2016). Safety of epidural corticosteroid injections. *Drugs in R&D*.

[77] Lee J. H., Lee S. H. (2016). Can repeat injection provide clinical benefit in patients with lumbosacral diseases when first epidural injection results only in partial response?. *Pain Physician*.

[78] Forst S. L., Wheeler M. T., Fortin J. D., Vilensky J. A. (2006). The sacroiliac joint: anatomy, physiology and clinical significance. *Pain Physician*.

[79] Fortin J. D., Tolchin R. B. (2003). Sacroiliac arthrograms and post-arthrography computerized tomography. *Pain Physician*.

[80] Kallewaard J. W., Geurts J. W., Kessels A., Willems P., van Santbrink H., van Kleef M. (2016). Efficacy, safety, and predictors of intradiscal methylene blue injection for discogenic low back pain: results of a multicenter prospective clinical series. *Pain Practice*.

[81] Peng B., Zhang Y., Hou S., Wu W., Fu X. (2007). Intradiscal methylene blue injection for the treatment of chronic discogenic low back pain. *European Spine Journal*.

[82] Leclaire R., Fortin L., Lambert R., Bergeron Y. M., Rossignol M. (2001). Radiofrequency facet joint denervation in the treatment of low back pain. *Spine*.

[83] Shanthanna H., Chan P., McChesney J., Paul J., Thabane L. (2014). Pulsed radiofrequency treatment of the lumbar dorsal root ganglion in patients with chronic lumbar radicular pain: a randomized, placebo-controlled pilot study. *Journal of Pain Research*.

[84] Ferrante F. M., King L. F., Roche E. A. (2001). Radiofrequency sacroiliac joint denervation for sacroiliac syndrome. *Regional Anesthesia and Pain Medicine*.

[85] Patel N., Gross A., Brown L., Gekht G. (2012). A randomized, placebo-controlled study to assess the efficacy of lateral branch neurotomy for chronic sacroiliac joint pain. *Pain Medicine*.

[86] Canovas Martinez L., Orduna Valls J., Parames Mosquera E., Lamelas Rodriguez L., Rojas Gil S., Dominguez Garcia M. (2015). Sacroiliac joint pain: prospective, randomised, experimental and comparative study of thermal radiofrequency with sacroiliac joint block. *Revista Española de Anestesiología y Reanimación*.

[87] Nikoobakht M., Yekanineajd M. S., Pakpour A. H., Gerszten P. C., Kasch R. (2016). Plasma disc decompression compared to physiotherapy for symptomatic contained lumbar disc herniation: a prospective randomized controlled trial. *Neurologia I Neurochirurgia Polska*.

[88] Bhagia S. M., Slipman C. W., Nirschl M. (2006). Side effects and complications after percutaneous disc decompression using coblation technology. *American Journal of Physical Medicine & Rehabilitation*.

[89] Alexandre A., Corò L., Azuelos A., Pellone M. (2005). Percutaneous nucleoplasty for discoradicular conflict. *Acta Neurochirurgica Supplement*.

[90] Barendse G. A. M., van Den Berg S. G. M., Kessels A. H. F., Weber W. E. J., van Kleef M. (2001). Randomized controlled trial of percutaneous intradiscal radiofrequency thermocoagulation for chronic discogenic back pain. *Spine*.

[91] Kapural L., Hayek S., Malak O., Arrigain S., Mekhail N. (2005). Intradiscal thermal annuloplasty versus intradiscal radiofrequency ablation for the treatment of discogenic pain: a prospective matched control trial. *Pain Medicine*.

[92] Urrútia G., Kovacs F., Nishishinya M. B., Olabe J. (2007). Percutaneous thermocoagulation intradiscal techniques for discogenic low back pain. *Spine*.

[93] D’Erme M., Scarchilli A., Artale A. M., Pasquali Lasagni M. (1998). Ozone therapy in lumbar sciatic pain. *La Radiologia Medica*.

[94] Bocci V., Borrelli E., Zanardi I., Travagli V. (2015). The usefulness of ozone treatment in spinal pain. *Drug Design, Development and Therapy*.

[95] Xiao-Feng H., Zhi-Jian Y., Gao-Jun T. (2003). Treatment of lumbar disc herniaton by using percutaneous intradiscal and paraspanal space injection of O_2_-O_3_ mixture. *Chinese Journal of Radiology*.

[96] Yue-Yong X., Jin-Lin T., Xiao Z., Deng-Ke L., Jia-Kai L. (2008). Treatment of discogenic back pain and invisible lumbar disc herniation with ozone ablation: a clinical study. *Chinese Journal of Interventional Imaging and Therapy*.

[97] Fu-Qiang C., Dan H., Fei S., Ping X., Deng-Bin A. (2007). Clinical study of percutaneous injection of intradisc and intervertebral foramen with ozone to treat lumbar disc herniation. *Pain Clinic Journal*.

[98] Qing-Hua Y., Guang-Jian Z., Ren-Shu L. (2015). Review of ozone in the treatment of painful diseases. *Journal of Cervicodynia and Lumbodynia*.

[99] Morales A., Yong R. J., Kaye A. D., Urman R. D. (2019). Spinal cord stimulation: comparing traditional low-frequency tonic waveforms to novel high frequency and burst stimulation for the treatment of chronic low back pain. *Current Pain and Headache Reports*.

[100] Kumar K., Taylor R. S., Jacques L. (2007). Spinal cord stimulation versus conventional medical management for neuropathic pain: a multicentre randomised controlled trial in patients with failed back surgery syndrome. *Pain*.

[101] North R., Shipley J., Prager J. (2007). Practice parameters for the use of spinal cord stimulation in the treatment of chronic neuropathic pain. *Pain Medicine*.

[102] Hayek S. M., Veizi E., Hanes M. (2015). Intrathecal hydromorphone and bupivacaine combination therapy for post-laminectomy syndrome optimized with patient-activated bolus device. *Pain Medicine*.

[103] Hong-Jun L., Xian-Zhong G., Wei-Yan L., Hong-Bin J., Yi J. (2014). Effects of dexmedetomidine on spinal morphine anaigesia in terminal cancer pain patients. *Chinese Journal of Pain Medicine*.

[104] Yong-Guo D., Long-Long Z., Cheng Z (2013). Treatment of 28 cases of lumbar disc herniation recurrence with silver needles. *Chinese Journal of Pain Medicine*.

[105] Zi-Long R., Nan-Chang S. (2017). Long-term efficacy of chronic lumbar muscle strain with silver acupuncture. *Modern Diagnosis & Treatment*.

[106] Hong-Kai J. (2017). Effect of silver needle in 22 cases of postoperative syndrome of lumbar intervertebral disc protrusion. *World Latest Medicine Information*.

[107] Cheng-Hong W., Hong-Fei C., Qian G., Hua C., Fu-Gen W. (2004). Observation on the effect of silver needle therapy in the treatment of failed back surgery syndrome. *Chinese Journal of Clinical Rehabilitation*.

[108] Cha-Di T., Xue-Dong S. (2012). Case report: 45 cases of third lumbar transverse process syndrome with piroxicam patch combined with small needle knife release. *Chinese Journal of Pain Medicine*.

[109] Song-Qing L., Bin W., Yong-Jie L., Zeng-Jing Y., Lei Z. (2014). Targeted injection combined with small needle knife in the treatment of third lumbar transverse process syndrome. *Chinese Journal of Traditional Medical Traumatology and Orthopedics*.

[110] Hu C., Wei-Ying C. (2015). Preliminary experience of the small knife in the treatment of the chronic soft tissue injuries. *Shanghai Medical and Pharmaceutical Journal*.

[111] van Middelkoop M., Rubinstein S. M., Kuijpers T. (2011). A systematic review on the effectiveness of physical and rehabilitation interventions for chronic non-specific low back pain. *European Spine Journal*.

[112] Holtzman S., Beggs R. T. (2013). Yoga for chronic low back pain: a meta-analysis of randomized controlled trials. *Pain Research and Management*.

[113] Macedo L. G., Bostick G. P., Maher C. G. (2013). Exercise for prevention of recurrences of nonspecific low back pain. *Physical Therapy*.

[114] Lam M., Galvin R., Curry P. (2013). Effectiveness of acupuncture for nonspecific chronic low back pain. *Spine*.

[115] Kumar S., Beaton K., Hughes T. (2013). The effectiveness of massage therapy for the treatment of nonspecific low back pain: a systematic review of systematic reviews. *International Journal of General Medicine*.

[116] Ebadi S., Henschke N., Nakhostin Ansari N., Fallah E., van Tulder M. W. (2014). Therapeutic ultrasound for chronic low-back pain. *The Cochrane Database of Systematic Reviews*.

[117] Buchmuller A., Navez M., Milletre-Bernardin M. (2012). Value of TENS for relief of chronic low back pain with or without radicular pain. *European Journal of Pain*.

[118] Wellington J. (2014). Noninvasive and alternative management of chronic low back pain (efficacy and outcomes). *Neuromodulation: Technology at the Neural Interface*.

[119] Oleske D. M., Lavender S. A., Andersson G. B. J., Kwasny M. M. (2007). Are back supports plus education more effective than education alone in promoting recovery from low back pain?. *Spine*.

[120] Wegner I., Widyahening I. S., van Tulder M. W. (2013). Traction for low-back pain with or without sciatica. *The Cochrane Database of Systematic Reviews*.

[121] Henschke N., Ostelo R. W., van Tulder M. W. (2010). Behavioural treatment for chronic low-back pain. *The Cochrane Database of Systematic Reviews*.

[122] Cherkin D. C., Sherman K. J., Balderson B. H. (2016). Effect of mindfulness-based stress reduction vs cognitive behavioral therapy or usual care on back pain and functional limitations in adults with chronic low back pain. *JAMA*.

[123] van Tulder M. W., Koes B., Seitsalo S., Malmivaara A. (2006). Outcome of invasive treatment modalities on back pain and sciatica: an evidence-based review. *European Spine Journal*.

[124] Ostelo R. W., van Tulder M. W., Vlaeyen J. W., Linton S. J., Morley S. J., Assendelft W. J. (2005). Behavioural treatment for chronic low-back pain. *The Cochrane Database of Systematic Reviews*.

[125] Kamper S. J., Apeldoorn A. T., Chiarotto A. (2014). Multidisciplinary biopsychosocial rehabilitation for chronic low back pain. *The Cochrane Database of Systematic Reviews*.

[126] Franke H., Franke J. D., Fryer G. (2014). Osteopathic manipulative treatment for nonspecific low back pain: a systematic review and meta-analysis. *BMC Musculoskeletal Disorders*.

[127] Heneweer H., Staes F., Aufdemkampe G., van Rijn M., Vanhees L. (2011). Physical activity and low back pain: a systematic review of recent literature. *European Spine Journal*.

[128] Hoy D., Brooks P., Blyth F., Buchbinder R. (2010). The Epidemiology of low back pain. *Best Practice & Research Clinical Rheumatology*.

[129] Linton S. J. (2000). A review of psychological risk factors in back and neck pain. *Spine*.

[130] Matsui H., Maeda A., Tsuji H., Naruse Y. (1997). Risk indicators of low back pain among workers in Japan. *Spine*.

[131] Su-Mei Z., Qiong X., Xue-Qiang W. (2012). Effect of health education on chronic lower back pain. *Chinese Journal of Rehabilitation*.

[132] Schaafsma F. G., Anema J. R., van der Beek A. J. (2015). Back pain: prevention and management in the workplace. *Best Practice & Research Clinical Rheumatology*.

